# Resting-state functional magnetic resonance imaging: the cornerstone of future neuroimaging

**DOI:** 10.1093/psyrad/kkaf032

**Published:** 2025-11-08

**Authors:** Jiaqi Jing, Chen Liu

**Affiliations:** Faculty of Psychology, Southwest University, Chongqing 400715, China; 7T Magnetic Resonance Imaging Translational Medical Center, Department of Radiology, Southwest Hospital, Third Military Medical University, Chongqing 400038, China

Over the past three decades, resting-state functional magnetic resonance imaging (rsfMRI) has evolved from an emerging concept to a widely used neuroimaging modality, offering unparalleled insights into the brain’s intrinsic functional architecture (Murphy *et al*., 2013). Unlike task-based fMRI paradigms, rsfMRI captures spontaneous brain activity when participants are not engaged in goal-directed behavior, enabling researchers to investigate spontaneous fluctuations in blood oxygen level-dependent (BOLD) signals and infer large-scale functional connectivity (Fox and Raichle, 2007). Since the seminal study by Biswal *et al*. (1995), which revealed functional connectivity between the left and right sensorimotor cortices at rest, rsfMRI has transitioned from skepticism to mainstream adoption, as evidenced by exponential growth in publications and the rise of large-scale initiatives such as the Human Connectome Project (HCP) (Van Essen *et al*., 2013) and the Adolescent Brain Cognitive Development (ABCD) (Casey *et al*., 2018). This historical progression and conceptual maturat ion have been comprehensively reviewed in Biswal & Uddin (2025), whose work further contextualizes the field’s evolution and future trajectory.

## Strengths and contributions

**Figure 1:**
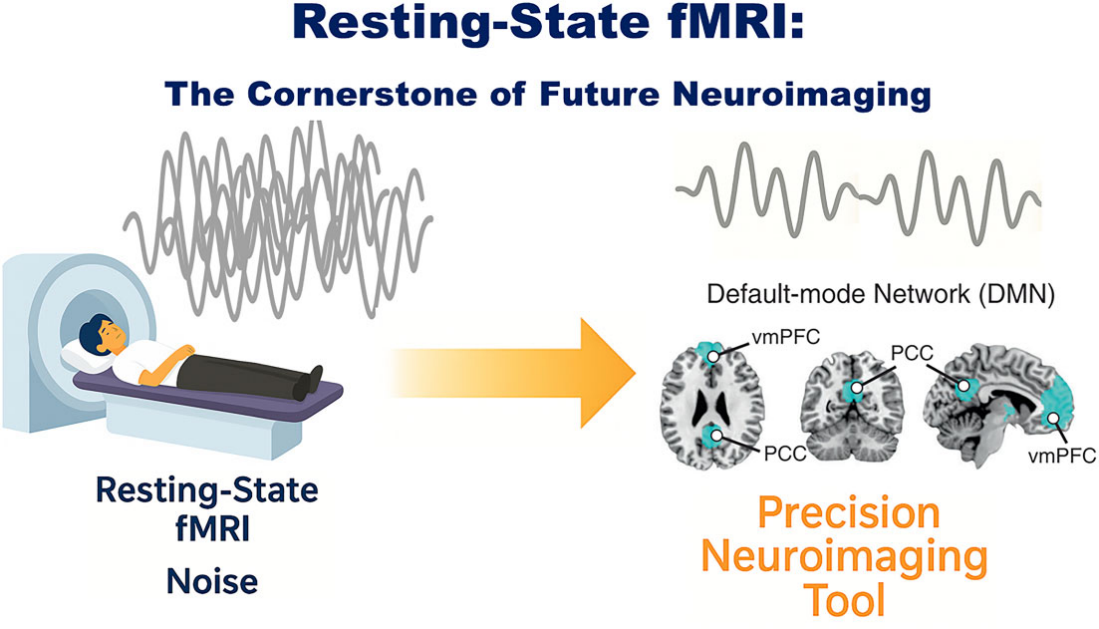
Resting-state fMRI: the cornerstone of future neuroimaging.

One of the most significant strengths of rsfMRI is its broad applicability and remarkable versatility. It is suitable for a wide range of populations, including those who are difficult to engage in tasks, such as pediatric (including fetal), clinical, and geriatric populations, since it does not rely on tasks and requires participants to rest for a relatively short time in the scanner. However, certain populations with severe intellectual disabilities or contraindications to MRI (e.g. metallic implants) remain challenging to study. At the same time, its utility extends across diverse application scenarios, ranging from clinical disease diagnosis and treatment prediction to basic animal experiments. For instance, it has been widely used to study psychiatric disorders including schizophrenia, bipolar disorder, autism spectrum disorders, and attention deficit hyperactivity disorders (ADHD) (Canario *et al*., 2021). Over the years, researchers have identified large-scale functional brain networks such as the default mode, dorsal attention network, visual network, motor network, and frontoparietal network, many of which closely resemble task-evoked activation patterns (Fig. 1). These discoveries have advanced our understanding of the brain functional organization across the lifespan.

Moreover, rsfMRI research has been driven by methodological innovations at the intersection of neuroscience, physics, and engineering and data-driven approaches such as independent component analysis (ICA), graph theory, and dynamic connectivity analysis. Importantly, rsfMRI has translational potential by enabling the prediction of cognitive traits and clinical outcomes based on individual-level functional connectivity patterns.

## Toward precision and clinical applications

In recent years, rsfMRI has evolved from a tool primarily employed in basic scientific research to one increasingly applied in clinical settings, particularly in the context of precision medicine and intelligent diagnostics. Within the therapeutic domain, connectivity maps of the default mode or salience networks have been applied in major depressive disorder (Gong *et al*., 2020) to guide individualized targets for neuromodulation techniques such as repetitive transcranial magnetic stimulation (rTMS) and deep brain stimulation (DBS), thereby improving treatment response (Downar *et al*., 2024; Xiao *et al*., 2024). In the realm of diagnosis and prognosis, rsfMRI-derived measures such as functional connectivity gradients are facilitating the identification of biomarkers that help distinguish pathological from healthy states, parse heterogeneity within disorders, and predict individual treatment responses (Hong *et al*., 2020; Jiang *et al*., 2023). Looking ahead, the emergence of artificial intelligence (AI) foundation models is poised to profoundly transform neuroimaging. Compared with task-based fMRI, rsfMRI offers broader applicability, a characteristic that aligns well with the data requirements of future foundation models. Consequently, the intelligent future of neuroimaging will probably rely heavily on rsfMRI data as a foundational resource, enabling the development of robust, generalizable models that span the diagnostic–therapeutic continuum in neurology and psychiatry.

## Limitations and persistent problems

Nevertheless, rsfMRI continues to face several persistent challenges. A major technical challenge is the difficulty of isolating neural signals from physiological and scanner-related noise, including motion, cardiac pulsations, and respiration (Uddin, 2020). The use of global signal regression (GSR), for example, continues to generate controversy: while it can reduce motion-related confounds, it may also artificially introduce negative correlations between brain regions (Murphy and Fox, 2017). Beyond these technical issues, the lack of standardized acquisition and preprocessing protocols, compounded by inconsistent network nomenclature, hampers comparability across studies (Uddin *et al*., 2023). From a clinical perspective, inadequate sample sizes in many studies compromise the generalizability of reported brain–behavior relationships, and machine learning models trained on limited datasets often fail to generalize across diverse populations (Marek *et al*., 2022). Furthermore, although new analytical approaches for rsfMRI data are continually emerging, their underlying physiological significance remains unclear (Cole *et al*., 2010). Therefore, greater validation and mechanistic insights from basic animal studies are needed; otherwise, the limited interpretability of rsfMRI will hinder its broader clinical translation and application. These challenges, however, also delineate clear priorities for the next phase of research.

## Future directions

In the future, there are several priorities that must be addressed to advance the field. First, recent work on developmental brain charts has served as a normative reference for quantifying individual variation in development, aging, and neuropsychiatric disorders (Bethlehem *et al*., 2022; Sun *et al*., 2025). Second, to enable broader clinical translation, it is essential to establish community-driven consensus standards similar to oncology practice guidelines (e.g. National Comprehensive Cancer Network), with regularly updated protocols that adapt to advances in MRI technology and provide tailored acquisition schemes for diverse clinical needs. Within this framework, optimizing scan duration is also critical: although longer scans improve reproducibility, they must be balanced against cost and participant burden, highlighting the need for practical guidelines (Ooi *et al*., 2025). Third, standardized pipelines will facilitate pooling of data from large-scale initiatives such as ABCD, HCP, and Autism Brain Imaging Data Exchange (ABIDE), thereby enhancing statistical power and reproducibility. Fourth, as rsfMRI is relatively easy to acquire, it is increasingly being adopted in large-scale datasets as training material for AI models, and its generalizability across disorders positions it as a strong candidate to serve as foundational data for the development of future neuroimaging foundation models. Finally, incorporating multimodal validation studies (e.g. combining BOLD fMRI with electrophysiology or calcium imaging) will be crucial for clarifying the neurophysiological basis of resting-state signals. Together, these directions position rsfMRI as a cornerstone for building the future of neuroimaging.

## Conclusion

In summary, rsfMRI has developed into an indispensable neuroimaging tool with distinct advantages for population studies and clinical translation. Its future impact will depend on resolving methodological inconsistencies and leveraging its unique scalability for AI-driven biomarker discovery. As the field moves toward precision neuroscience, rsfMRI is poised to serve as the cornerstone for building generalizable, clinically actionable models of brain function.
